# Assessing the competency of pharmacists in writing effective curriculum vitae for job applications: a cross-sectional study and readability index evaluation

**DOI:** 10.1186/s12909-023-04870-5

**Published:** 2023-11-20

**Authors:** Mohanad Odeh, Muna Oqal, Hanan AlDroubi, Basem Al-Omari

**Affiliations:** 1 Department of Clinical Pharmacy and Pharmacy Practice, Faculty of Pharmaceutical Sciences, The Hashemite University, P.O. Box 330127, Zarqa, 13133 Jordan; 2 Department of Pharmaceutics and Pharmaceutical Technology, Faculty of Pharmaceutical Sciences, The Hashemite University, P.O Box 330127, Zarqa, 13133 Jordan; 3 Department of Public Health and Epidemiology, College of Medicine and Health Sciences, Khalifa University of Science and Technology, P O Box 127788, Abu Dhabi, United Arab Emirates

**Keywords:** Curriculum vitae, Readability index, Personal statement, Cover letter, Pharmacists’ job application

## Abstract

**Background:**

In today’s competitive job market, pharmacists must have a well-crafted curriculum vitae (CV), cover letter, and personal statement. However, non-native English speakers may face challenges in crafting effective job application documents. Jordan is one such country where English is a second language for many, and little is known about the CV/job application writing skills of Jordanian pharmacists. Therefore, this study examined Jordanian pharmacists’ ability to write job applications cover letters, and personal statements in English and investigated the association between several demographics and professional variables and the readability index of cover letters and personal statements.

**Methods:**

This study aimed to investigate Jordanian pharmacists’ ability to write job applications cover letters, and personal statements in English and evaluate the readability of their personal statements and cover letters. The data were blindly and independently reviewed by two researchers. The readability of the cover letters and personal statements was assessed using an online calculator that assigns a readability index score. A readability score of 7–12 was considered “target”, while scores above 12 or below 7 were considered “complicated” or “simple”, respectively. The relationship between readability index scores and other variables was analyzed using the chi-square test with a statistical significance level of 0.05.

**Results:**

The study recruited 592 pharmacists. Most applicants, specifically 62.3%, were female, and 60.0% of them graduated more than six months before submitting their job applications. While 78.2% of the applications included a personal statement, only 34.8% included a cover letter, and 27.2% provided both. Of the 206 cover letters written in English, 43.2% were tailored, and 80.6% were structured. The study also found that the provision of an official photo was associated with providing a cover letter (*P* < 0.001, Phi(φ) = 0.14) while providing a structured cover letter was associated with including a personal statement (*P* < 0.001, Phi (φ) = 0.24). Only 102 cover letters and 65 personal statements had readability index scores within the target range.

**Conclusion:**

In this study, most Jordanian pharmacists undervalue the importance of cover letters and personal statements and lack job application writing skills. The study also highlighted the need for improved pharmacists’ English proficiency to write effective job application documents in Jordan.

## Introduction

Over the past decade, the world encountered a significant increase in the number of graduated pharmacists [1]. Jordan graduates almost 2000 pharmacists every year [2]. This is matched by the dramatic increase in the number of pharmacy schools in Jordan from two public schools in early 1990 to 18 (five public and 13 private) in 2023 [3]. An analysis of the global pharmacy workforce capacity in 2016, showed that the density of pharmacists per 10,000 population in Jordan was 22.5 compared to the average world mean of 6.02 pharmacists per 10,000 population [1]. Furthermore, Jordan is considered to have the third-highest density of pharmacists after Malta and Japan [1, 4]. For pharmacists to be able to compete for jobs in such high density, their profiles must be presented and perceived at a highly skilled and qualified level [5]. Furthermore, pharmacists need to be equipped with a broad knowledge of professional career skills, such as curriculum vitae (CV) development which is a primary step in applying for a job [6].

CV originated from Latin and means “course of life”. Therefore, it should provide a comprehensive overview of education, skills, interests, and other professional and personal information [7–9]. Moreover, a CV should be an eye-catching document, tailored for pursuing a new job, and created by professionals to give a summary of their skills and achievements [8]. For pharmacists, there is a consensus on which elements of the CVs are required, such as personal information, education, work experiences, presentations, publications, awards licensure, certifications, and pharmacy registration [10, 11]. In particular, the two main parts that should be crafted skillfully and carefully are the personal statement [12, 13] and the cover letter that is usually submitted with the job application [8, 14].

In countries where English is a second language, such as Jordan, writing CVs and cover letters brings additional challenges that require more training [15]. This is especially important as the Jordanian Ministry of Higher Education (MHE) stated that English is the official language for studying medical sciences including pharmacy, and It is the main language used in professional communication within the pharmaceutical sectors in Jordan [16]. Therefore, it is a requirement that CVs for job applications should be written in English. Generally, several ideas and variables are discussed within the cover letter [17, 18]. In all cases having a well-written, tailored to the purpose, well-organized, and structured cover letter and personal statement is vital for pharmacists’ job applications [8, 14, 17, 19].

Generally, in the Middle East region, medical representatives (also referred to as pharmaceutical representatives or drug representatives) are predominantly pharmacists. The primary roles of medical representatives are to establish and maintain communication between drug stores and healthcare providers and to promote the pharmaceutical products of the drug stores [20, 21]. Local data indicated that at least 35% of graduate pharmacists in Jordan are working as medical representatives [22]. Medical representatives receive highly ambitious salaries making this job widely popular and competitive in the Middle East [23, 24]. Therefore, pharmacists must understand the criteria for evaluating job applications. One of the most used methods to evaluate the quality of cover letters and personal statements is the English written quality and subsequently the readability index assessment [8, 9, 11, 13, 14]. The readability index or readability score is an estimation of how difficult it is to read the text. This estimation is made by quantifying the text’s complexity by measuring attributes of the text such as word counts, sentence lengths, and syllable counts [25, 26]. Over 90 years of research, tens of readability formulas have been suggested and extensively studied by literature, mainly by linguistic specialists for school teachers, print media, and other purposes [25].

Recently, readability measures have gained more attention in health-related fields, mainly for patient education [26–29]. There is a limited number of published studies investigating aspects related to CVs in a scientific objective approach. Furthermore, to the best of our knowledge, there are no studies analyzing pharmacists’ cover letters and personal statements based on the readability index measures. Therefore, this study examines Jordanian pharmacists’ ability to write job applications cover letters, and personal statements in English and investigates the association between several demographics and professional variables and the readability index of cover letters and personal statements.

## Methods

### Population, setting, and study design

This cross-sectional study recruited Jordanian pharmacists (bachelor’s degree in pharmacy or Doctor of Pharmacy) applying for a medical representative job at a large international drug store in Jordan. The drug store contacted all Job applicants by phone to confirm their consent to participate in the study. Applicants were reassured that personally identifiable information would not be shared with the researchers and that the participants’ identities remained anonymized. Furthermore, applicants were informed that the decision regarding the participant in the study has no impact on the recruitment process. Those who provided consent were considered for inclusion in the current study. Participating CVs/job applications were made anonymous by the drug store, through a simple anonymization process, as a numeric identification (ID) code was assigned to each applicant’s job application, and any information that could identify the participants such as names, phone numbers, and addresses were removed from the applications. The anonymized CVs/job applications with the ID codes were received by the primary investigator (MO) who completed a further assessment of the anonymity. Two senior managers from the drug store had reviewed the anonymization process and they have the master matching list between ID codes and the Original CVs/job applications.

Pharmacists’ CVs/job applications were included if they were written in English and submitted electronically by e-mail to the drug store. CVs/job applications were excluded if they were written in Arabic, for non-pharmacists or diploma in pharmacy, were incomplete, or were submitted as scanned documents of hard copies that were not possible to transfer without being rewritten.

### Ethical consideration

Ethical approval was obtained from the research ethics committee/institutional review board (IRB) of Hashemite University, Zarqa, Jordan, with reference number (14/9/2021/2022).

### Sample size calculations

With the reference that the number of pharmacists in Jordan is 29,463 pharmacist [30] when considering a confidence level of 95%, and a margin of error of 5%, as recommended by Taherdoost [31], the sample size calculator [32] determined that a representative sample of 381 participants are required for this study.

### Data collection

Data were collected between the 1st of February 2022 and the 30th of April 2022. All eligible applications that met the inclusion criteria were reviewed by a research assistant. The following data were inserted into an Excel sheet: gender, newly graduated (within six months of submitting the application) or not, whether the CV/job application included a cover letter or not, the cover letter was tailored to the pharmacist job in a drug store or not, a personal statement was provided or not, the fonts and punctuations were consistent or not, a list of experience was provided or not, a CV template was used or not, CV/job application number of pages, CV/job application file format, type of personal photo, marital status, city of living, a list of skills, university grade level, and referees list.

Each CV/job application data was extracted by two researchers, assessed the inclusion of the above variables, and categorized the cover letters as tailored and structured. The two researchers extracted the data blindly and independently of each other. The principal investigator (PI) examined the data extracted by both researchers for consistency. Inconsistency was primarily discussed by both researchers and the PI resolved any further disagreement. A cover letter was considered tailored if it addressed the announced job description by having key terms related to the medical representative tasks or the job advertised. The cover letter was considered structured if it had an introductory paragraph, a body, and a closing paragraph.

### Data analysis

Descriptive statistics were used to summarize frequencies. Percentages and numbers were used for categorical variables using Microsoft Excel. The cover letter and personal statements documents were transferred to the readability online calculator (StoryToolz), which analyses the surface characteristics of a document, including sentence length and other readability measures [33]. The readability online calculator provided readability scores for each document using the following formulas: Flesch Kincaid grade level, automated readability index, Coleman Liau, Gunning Fog index, and simple measure of Gobbledygook (SMOG) index [25, 26], then the average grade level category was calculated [33]. The readability index scores were categorized as “simple” if the score is less than 7, “target” if the score is between 7 and 12, and “complicated” if the score was more than 12 [29, 34, 35].

The SPSS software package (version 24.0) was used to examine the relationship between predictor variables and readability scores. The association between the nominal (categorical) variables was assessed using the chi-square test. The formula used to calculate degrees of freedom (df) for the chi-square test was: df = (r − 1) × (c − 1), where “r” represents the number of categories within the demographic variable, and “c” represents the number of options related to each category. To measure the strength of association of a nominal-by-nominal relationship (a measure of effect size), Phi (Φ) factor or Cramer’s V coefficient when having more than two dichotomous variables was calculated and illustrated. The values of ‘1’ indicate a complete association, ‘0’ indicates no association, ‘0.1 indicates a small association, ‘0.3’ indicates a medium association, and ‘0.5’ indicates a large association. Phi (Φ) or Cramer’s V coefficient was only illustrated when there was a statistical significance (*P*-value of less than 0.05).

## Results

### Descriptive results

In total, 1322 applications were submitted to the drug store, and 950 applicants accepted to be considered for this study. The 950 job applications were screened against the inclusion/exclusion criteria, and 358 CV/job applications were excluded for the following reasons: non-pharmacist job applications (n = 207, 57.8%), job applications written in Arabic (n = 84, 23.5%), diploma in pharmacy (n = 39, 10.9%), hard copies or scanned CVs/job applications (n = 23, 6.4%), and incomplete applications (n = 5, 1.4%). Finally, 592 CVs/job applications were included in the final analysis.

Most CVs/job applications were for female applicants (62.3%), not newly graduated (60.0%), used professional CV template (54.1%), consistently used the same font (92.4%), reported the city of residence (86%), and a list of previous work experience (64.0%). Furthermore, most CVs/job applications did not include the marital status of the applicants (60.8%), did not use appropriate punctuation (54.6%), and did not provide a list of referees (89%).

The personal statement was included in 78.2%, with personal photos (81.1%). Most participants did not provide a cover letter (65.2%). Of those 206 who provided a cover letter written in English, it was tailored in 43.2% and it was structured in 80.6%. The list of skills was provided in 94.6% of the applications. However, only 23.2% of those who provided the list of skills have their skills tailored to the advertised Job (see Table 1).

**Table 1:** Descriptive information and demographic variables

CV/job application variable	Levels	n = 592 (%)
Gender	Female Male Not provided	369 (62.3%) 213 (36%) 10 (1.7%)
Newly graduated	No Yes Not provided	355 (60%) 227 (38.3%) 10 (1.7%)
CV template	Regular template Professional template	272 (45.9%) 320 (54.1%)
The same font used consistently	Yes No	547 (92.4%) 45 (7.6%)
Punctuations used consistently	Yes No	296 (45.4) 323 (54.6%)
Experience list included	Yes No Incomplete	379 (64.0%) 203 (43.3%) 10 (1.7%)
Marital status included	Yes No	232 (39.2%) 360 (60.8%)
City of living included	Yes No	509 (86%) 83 (14%)
Personal statement included	Yes No	463 (78.2%) 129(21.8%)
The list of skills included	Yes No	560 (94.6%) 32 (5.4%)
List of skills tailored to the advertised job (n = 560)	Yes No	130 (23.2%) 430 (76.8%)
Personal photo included	Yes No	480 (81.1%) 112 (19.9%)
Type of personal photo	Casual Official No photo provided	129 (21.8%) 351 (59.3%) 112 (18.9%)
University grade level	Excellent Very Good Good Satisfactory Not provided	94 (16%) 140 (24%) 94 (16%) 3 (0.005%) 261 (44%)
Referees list included	Yes No	65 (11.0%) 527 (89.0%)
At least one misuse of big and small letters	Yes No	466 (78.7%) 126 (21.3%)
CV file format	PDF Word Image Other	486 (82.1%) 82 (13.9%) 16 (2.7%) 8 (1.4%)
Number of CV/job application pages	1 2 3 4 5 6	307 (51.9%) 217 (36.7%) 50 (8.4%) 14 (2.4%) 3 (0.5%) 1 (0.2%)
Provided a cover letter in English	Yes No	206 (34.8%) 386 (65.2%)
**English cover letter (n = 206)**		
Tailored	Yes No	89 (43.2%) 117 (56.8%)
Structured	Yes No	166 (80.6%) 40 (19.4%)

### Cover letters and personal statements readability index

Of the 206 participants who submitted a cover letter, 49.5% had readability index scores within the target category, 4.3% had readability index scores within the simple category, and 26.2% had readability index scores within the complicated category (see Table 2).

Seven of the 463 participants who completed personal statements were excluded, as it was not possible to copy the personal statement to the readability score software. The majority of the 456 included personal statements had readability index scores within the complicated category (82.2%), 16.9% had readability index scores within the target category, and 0.9% had readability index scores within the simple category (see Table 2).

**Table 2:** Cover letter and personal statements’ readability index scores matched with readability formulas and their average

Readability index scores	Flesch Kincaid grade level category	Automated readability index category	Coleman Liau category	Gunning fog index category	SMOG index category	Average grade level n = 206
**Cover letter readability index scores**						
Less than 7 (Simple)	80 (38.8%)	82 (39.8%)	50 (24.3%)	35 (17%)	32 (15.5%)	50 (24.3%)
7 to 12 (Target)	97 (47.1%)	78 (37.9%)	114 (55.3%)	77 (37.4%)	111 (53.9%)	102 (49.5%)
More 12 (complicated)	29 (14.1%)	46 (22.3%)	42 (20.4%)	94 (45.6%)	63 (30.6%)	54 (26.2%)
**Personal statement readability index scores**						
Less than 7 (Simple)	9 (2%)	4 (0.9%)	2 (0.4%)	2 (0.4%)	3 (0.7)	4 (0.9%)
7 to 12 (Target)	126 (27.6%)	95 (20.8%)	111 (24.4%)	39 (8.6%)	65 (14.3%)	77 (16.9%)
More 12 (complicated)	321 (70.4%)	357 (78.3%)	343 (75.2)	415 (91.0%)	388 (85.0%)	375 (82.2%)

### The association between providing a cover letter and other variables

The statistically significant association for “providing cover letter” was with “type of personal photo” (*P* < 0.001), “University grade level” (*P* = 0.027), and “CV file format” (*P* = 0.001). Other variables did not have a statistically significant association with “providing a cover letter” (see Table 3). Testing the strength of association of a nominal-by-nominal relationship was only applicable to the ‘’ type of personal photo’ (Phi(φ) = 0.14).

**Table 3:** The association between providing a cover letter and other variables

Variable	Levels	Provide cover letter		*p*-value chi-square
		Yes (n = 206)	No (n = 386)	
Gender	Female Male Not provided	129 75 2	240 138 8	0.61
Newly graduated	No Yes Not provided	132 72 2	223 155 8	0.246
The same font used consistently	Yes No	185 21	362 24	0.082
Punctuations used consistently	Yes No	102 104	221 165	0.07
Experience list included	Yes No Incomplete	75 126 2	125 253 8	0.28
Marital status included	Yes No	85 121	147 239	0.45
City of living included	Yes No	177 29	332 54	0.98
Personal statement included	Yes No	161 45	302 84	0.98
The list of skills included	Yes No	182 14	368 18	0.28
List of skills tailored to the advertised job	No list of skills* Yes No	14 40 152	18 90 278	0.34
Personal photo included	Yes No	172 34	308 78	0.273
Type of personal photo	Casual Official No photo*	29 143 34	100 208 78	< 0.001 Phi(φ) = 0.14
University grade level	Excellent Very Good Good Satisfactory Not provided	41 55 20 1 89	53 85 74 2 172	0.027**
Referees list included in the CV/job application	Yes No Upon request	29 130 47	36 262 88	0.2
At least one misuse of big and small letters at the start of the sentences	Yes No	192 14	354 32	0.518
At least one misuse of big and small letters for cities and names	Yes No	201 5	363 23	0.054
CV file format	PDF Word Image Other	186 18 2 0	300 64 14 8	0.001^**^
Number of CV/job application pages	1 2 3 4 5 6	92 84 24 4 2 0	215 133 26 10 1 1	0.11

*Not included in the cross-tabulation chi-squared test. **Fisher exact test used as one or more cells have an expected count of less than five, Phi was not calculated

### The association between providing personal statements and other variables

Providing personal statement had a statistically significate association with “CV template” (*P* < 0.001), “same font used consistently” (*P* = 0.002), “skills list included” (*P* < 0.001), “referees list included” (*P* = 0.047), “CV file format” (*P* < 0.001), and “structured cover letter provided” (*P* < 0.001). Other variables did not have a statistically significant association with “providing personal statement” (see Table 4). Testing the strength of association of a nominal-by-nominal relationship revealed that the strongest association was with “structured cover letter provided” (Phi (φ) = 0.24) and the least with “referees list included” (Phi (φ) = 0.10).

**Table 4:** Association between providing personal statement and other variables

Variable	Levels	Provide personal statement		*P*-value Chi-square
		Yes (n = 463)	No (n = 129)	
Gender	Female Male Not provided*	295 168 0	74 45 10	0.75
Newly graduated	No Yes Not provided*	274 189	81 38 10	0.08
CV template	Regular template Professional template	192 271	80 49	< 0.001 Phi(φ) = 0.17
The same font used consistently	Yes No	436 27	111 18	0.002 Phi(φ) = 0.12
Punctuations used consistently	Yes No	258 205	65 64	0.28
Experience list included	Yes No Incomplete	294 169 0	85 34 0	0.1
Marital status included	Yes No	178 285	54 75	0.48
City of living included	Yes No	385 78	104 25	0.51
The list of skills included	Yes No	448 15	112 17	< 0.001 Phi(φ) = 0.18
List of skills tailored to the advertised job	No list of skills* Yes No	15 108 340	17 22 90	0.32
Cover letter included	Yes No	161 302	45 84	0.98
Personal photo included	Yes No	381 82	99 30	0.15
Type of personal photo	Casual Official No photo*	108 273 82	21 78 30	0.38
University grade level	Excellent Very Good Good Satisfactory Not provided	76 119 73 2 192	18 21 21 1 68	0.18
Referees list included	Yes No Upon request	49 298 116	16 94 19	0.047 Phi(φ) = 0.10
CV file format	PDF Word Image Other	387 65 11 0	99 17 5 8	< 0.001^**^
Number of CV/job application pages	1 2 3 4 5 6	249 157 41 11 3 1	57 60 9 3 0 0	0.24
Provide a cover letter in English	Yes No	160 303	46 83	0.82
A structured cover letter provided	No cover letter* Yes No	303 32 128	83 38 8	< 0.001 Phi(φ) = 0.24

*Not included in the cross-tabulation chi-squared test. **Fisher exact test used as one or more cells have an expected count of less than five, Phi was not calculated

### The association between the cover letter readability index and other respondents’ variables

Gender, including experience list, and providing a tailored cover letter had a statistically significant association with the cover letter readability index. Females submitted more complex cover letters with higher readability index scores than males (*P* = 0.021). Applicants with no “experience list included” had more complex cover letters than those who provided a list of experiences (*P* = 0.017). Furthermore, the highest statistically significant association with the cover letter readability index was with providing a tailored cover letter (*P* < 0.0001). Most applicants who provided cover letters had the target readability index (see Table 5). Testing the strength of association of a nominal-by-nominal relationship revealed that the strongest association was between the readability index and providing a tailored cover letter (strength of association = 0.36), followed by both “gender” and “experience list included” (strength of association = 0.24).

**Table 5:** Association between cover letter readability index and participants’ characteristics

Variable (n = 206)	Level	Cover letter readability index			X^2^	Strength of association	*P*-value
		Less than 7	7 to 12	More 12			
Gender	Female, 129 Male, 75 Not provided,2	25 (19.4%) 23 (30.7%) 2 (100%)	64 (49.6%) 38 (50.7) 0	40 (31%) 14 (18.7%) 0	11.56	0.24	0.021
Newly graduated	Yes,72 No,132 Not provided, 2	15 (20.8) 33 (25) 2 (100)	33 (45.8) 69 (52.3) 0	24 (33.3) 30 (22.7) 0	9.02	0.21	0.061
Experience list included	Yes, 126 No, 78 Incomplete, 2	36 (28.6) 12 (15.4) 2 (100)	62 (49.2) 40 (51.3) 0	28 (22.2) 26 (33.3) 0	12.1	0.24	0.017*
Skills List included	Yes, No,	96 (50) 3 (25)	45 (23.4) 6(50)	51 (26.6) 3 (25)	6.3	0.12	0.18
University grade level	Excellent, 41 Very Good, 55 Good, 20 Satisfactory, 1 Not provided, 89	4 (9.8) 12 (21.8) 5 (25) 0 29 (32.6)	21 (51.2) 33 (60) 9 (45) 1 (100) 38 (42.7)	16 (39) 10 (18.2) 6 (30) 0 22 (24.7)	13.5	0.17	0.095
Tailored	Yes, 89 No, 117	7 (7.9) 43 (36.8)	48 (53.9) 54 (46.2)	34 (38.2 20 (17.1)	26.59	0.36	< 0.0001
Structured	Yes, 164 No, 42	36 (22) 14 (33.3)	85 (51.8) 17 (40.5)	43 (26.2) 11 (26.2)	2.65	0.11	0.27

*Not included in the cross-tabulation chi-squared test

### The association between the personal statement readability index and other respondents’ variables

There were no statistically significant associations between the personal statement readability index and other respondents’ characteristics (see Table 6).

**Table 6:** Association between personal statement readability index and participants’ characteristics

Variable (n = 206)	Level	Personal statement readability index			X^2^	strength of association	*P*-value
		Less than 7	7 to 12	More 12			
Gender	Female, 291 Male, 165 Not provided, 190	1 (0.3) 3 (1.8)	51 (17.5) 26 (15.8)	239 (82.1) 136 (82.4)	2.8	0.078	0.25
Newly graduated	Yes, 186 No, 270	2 (1.1) 2 (0.7)	37 (19.9) 40 (14.8)	147 (79) 228 (84.4)	2.2	0.07	0.33
Experience list included	Yes, 288 No, 168	3 (1) 1 (0.6)	45 (15.6) 32 (19)	240 (83.3) 135 (80.4)	1.1	0.05	0.58
Skills List included	Yes, No,	73 (16.6) 0 (0)	4 (0.9) 4 (26.7)	364(82.5) 11 (73.3)	1.2	0.05	0.56
University grade level	Excellent, 76 Very Good, 116 Good, 72 Satisfactory, 2 Not provided, 190	0 1 (0.9) 1 (1.4) 0 2 (1.1)	15(19.7) 19 (16.4) 10 (13.9) 1 (50) 32(16.8)	61(80.3) 96 (82.8) 61 (84.7) 1(50) 156 (82.1)	3.6	0.09	0.96
Tailored Cover letter	No cover letter* Yes, No,	0 1 (1.3) 3 (3.8)	51 13(16.5) 13 (16.2)	246 65 (82.2) 64 (80)	1.00	0.06	0.61
Structured Cover letter	No cover letter* Yes, No,	0 2(1.6) 2 (6.3)	51 19 (15) 7(21.9)	246 106 (83.5) 23 (71.9)	3.4	0.08	0.18

*Not included in the cross-tabulation chi-squared test

## Discussion

This study included 592 participants. While the cover letter is an important document that introduces the CVs/job applications [14, 36], only 206 out of the 592 participants included English cover letters, 89 of the cover letters were tailored, and 166 were structured. Furthermore, less than half of the cover letters (17.2% of the total participants) had readability index scores within the target category (score of 7–12) and females submitted more complex cover letters with higher readability index scores than males. Generally, gender had been identified as a factor that influences the readability index for paragraphs written by Jordanian students; female students had higher readability than males. The readability index has been widely used to measure textual difficulties [25] in education and assessment of the English written language [8, 9, 11, 13, 14]. Readability is concerned with the document layout and comprehensibility or understandability of written texts [37]. Therefore, these results suggest that more than 50% of the participants who provided cover letters (n = 206) had poor layout and comprehensibility cover letters.

Over 78% of the participants included personal statements and over 94% provided lists of skills. However, only 23% of the participants who provided lists of skills had these skills tailored to the advertised Job. The personal statement is an important document to persuade the reader to positively consider the qualifications, key traits, experiences, and aspirations of the applicant [38], and provide the applicants with the opportunity to highlight the most important skills and talents [7, 11, 14]. Taking into consideration that less than 22% of the total participants customized the personal statement to the advertised job indicates that participants may have been using fixed templates for the personal statements and misjudged the value of the personal statements to highlight their experience. However, further investigation and interviews with participants may assist in determining the reasons. The readability index of the personal statements confirmed the poor layout and comprehensibility of the participants’ job applications, as less than 17% of the participants who provided personal statements (n = 456) had readability index scores within the target category (score of 7–12).

Our results showed that providing a cover letter with the participants’ application had a statistically significant association with the type of personal photo, University grade level, and CV file format. Participants who submitted official type photos in PDF format CV and did not provide the University grade levels are more likely to provide cover letters as part of their job application than others. To date, there is no evidence to explain this association. The value of providing a photo with the job application has been argued with no consensus agreement and the format of the CV file and providing the University grade levels have not been discussed in the literature. However, it is feasible to propose that applicants who provided an official type of photo in PDF format CV may have a better understanding of the value of the cover letter, and those who did not provide the University grade levels allowed themselves to explain these grades in the cover letter. Furthermore, more factors had a significant association with providing a personal statement than providing a cover letter. Participants who used a professional CV template used the same font consistently, included a list of skills, did not include a referees list, submitted a PDF format CV, and did not provide a structured cover letter are more likely to provide personal statements as part of their job application than others. These findings emphasize our suggestion that those who provide a cover letter and personal statements (approximately 27.2 of the total participants (n = 592)) have satisfactory knowledge of how to write job applications for pharmacists’.

The findings of our research are not surprising, especially since the English language is a second language in Jordan. These issues of cover letters and personal statement writing exist even in countries where English is the native language. According to CareerBuilder survey 61% of hiring managers automatically dismiss a candidate with spelling and typo errors in their job applications [39]. Those managers identified the reasons for instant rejections of the job applications to be spelling mistakes, copying wording from the job ad, and inappropriate email addresses [39]. Therefore, applicants must consider these points during the job application process to avoid early rejection. Furthermore, a recent report by Andrew Hunter confirmed that in the United Kingdom (UK) more than 9 out of 10 CVs contain spelling errors [40]. Although the principal ruling is that the candidates did not make sufficient effort for the application process, this point is controversial. It can be argued that applicants may have been relying on their spell checker which was disabled. More importantly, others may debate that applicants who had mini typos could be the perfect candidate for the job [39]. However, taking into consideration that some job applications may end up in the shredder due to minor typos or grammatical mistakes, the applicant should pay attention to these points to maximize their opportunities for an interview. Although the CareerBuilder survey and Andrew Hunter’s are based on native English-speaking countries, they may indicate the mentality of recruiters in general. Especially, since English is the official language used in pharmaceutical professional communication in Jordan [16].

There is an increasing demand to integrate skills like professional communication and teamwork coordination [41], virtual skills and digital proficiency [42], Innovation skills [43], and professional interpersonal communication [3, 44], in addition to professional writing for both pharmacists and students. Although there is limited literature on assessing students’ retention of career skills knowledge and their perceived importance, a recent study confirmed that tailored workshops notably improve awareness of these skills [6]. Additionally, other studies endorsed the practical benefits gained through mastering virtual skills [42], and collaborative communication [41].

The findings of our study indicate that most Jordanian pharmacists do not submit a strong job application that includes vital components such as cover letters and personal statements. Therefore, the paper highlights the importance of integrating these skills within pharmacy curriculums to enhance alumni students’ career applications and developments.

### Strengths and limitations

To our knowledge, this is the first study investigating the pharmacists’ cover letters and personal statements writing skills for job applications using the readability index and examining the association between the readability index and other variables. The findings highlight several uninvestigated areas such as pharmacist cover letters and personal statements writing skills and the relationship between several factors and the readability index of these two documents. Despite these important strengths, this study has some limitations, such as the lack of qualitative data to determine the reasons behind Jordanian pharmacists’ limited job application writing skills. Therefore, future studies should include qualitative analysis of participant interviews to bridge this gap. In spite of the quantitative benefits of using readability indices, a general limitation of the readability indices is that they cannot judge the cohesion, clarity of the meaning, vocabulary quality, and understandability [25, 26, 28, 29].

## Conclusion

In our study, most Jordanian pharmacists did not provide English cover letters or personal statements and very few pharmacists provided tailored or structured cover letters for job applications. Our findings suggest that providing a cover letter and personal statement may indicate satisfactory job application writing knowledge and skills. However, most applicants who provided these documents had simple or complicated readability indices, highlighting the lack of cover letters and personal statements writing skills among Jordanian pharmacists. Our study highlights the need for pharmacist educators to integrate CV and job application writing skills within pharmacy curriculums to enhance alumni career applications and development. By doing so, pharmacy students will be better prepared to highlight their skills and competencies in job applications, ultimately leading to better job opportunities and a stronger pharmacy workforce.

## Data Availability

The datasets used and/or analyzed during the current study are available from the corresponding author upon reasonable request.
